# Preparation of Aditoprim Injection against *Streptococcus suis* in Pigs and a Dose Regimen Based on Pharmacokinetic-Pharmacodynamic Modeling

**DOI:** 10.3390/pharmaceutics14040730

**Published:** 2022-03-28

**Authors:** Wei Qu, Mengxiao Dong, Yuanhu Pan, Shuyu Xie, Zonghui Yuan, Lingli Huang

**Affiliations:** 1 National Reference Laboratory of Veterinary Drug Residues (HZAU), Huazhong Agricultural University, Wuhan 430070, China; qw@mail.hzau.edu.cn (W.Q.); dongmx2022@mail.hzau.edu.cn (M.D.); panyuanhu@mail.hzau.edu.cn (Y.P.); xieshuyu@mail.hzau.edu.cn (S.X.); yuan5802@mail.hzau.edu.cn (Z.Y.); 2 MAO Key Laboratory for Detection of Veterinary Drug Residues, Huazhong Agricultural University, Wuhan 430070, China; 3 MOA Laboratory for Risk Assessment of Quality and Safety of Livestock and Poultry Products, Huazhong Agricultural University, Wuhan 430070, China

**Keywords:** aditoprim, injection, *Streptococcus suis*, pharmacokinetics-pharmacodynamics modeling, alveolar fluid

## Abstract

In order to effectively treat the infection of *Streptococcus suis* and reduce the emergence of drug-resistant bacteria, an aditoprim (ADP) injection was developed in this study. The pharmaceutical property investigation results demonstrated that ADP injection was a clear yellow liquid with 10 g ADP distributing in every 100 mL solution uniformly. Its pH value and drug content were around 6.20 and 99.35~100.40%, respectively. And quality assessment preliminarily indicated its reliable quality and stability. Additionally, the bronchoalveolar lavage fluid method was first applied to evaluate accurate ADP concentration at infection site in this study. Through pharmacodynamic assay, the MIC, MBC and MPC of ADP against *Streptococcus suis* CVCC 607 was 2 μg/mL, 4 μg/mL and 12.8 μg/mL, respectively. The bacteria growth inhibition curves showed that ADP was a concentration-dependent antibacterial drug, and the PK-PD model parameter of AUC/MIC was selected. The pharmacokinetic parameters of alveolar fluid evaluated by WinNonlin software revealed similar pharmacokinetic process of ADP in healthy pigs and infected pigs. Combined with pharmacokinetics-pharmacodynamics (PK-PD) modeling, the dosage regimen of 3~5 days with an interval of 12 h at 4.10 mg/kg or 5.91 mg/kg could be adopted to treat the infection of *Streptococcus suis*. Consequently, this ADP injection with a multi-dose protocol would be a promising antimicrobial product for efficient treatment of *S. suis* infection of pigs.

## 1. Introduction

Swine streptococcosis is one of the most important zoonosis for pig breeding industry worldwide. Its morbidity is about 30~70%. And its mortality is 10~30%, which will rise to 80~90% without proper treatment [1]. *Streptococcus suis* (*S. suis*) is the pathogen causing the disease, and its pathogenesis is complex and not quite clear yet. The antibiotics for the treatment of streptococcosis mainly contain β-lactams, tetracyclines, macrolides, sulfonamides and quinolones. However, increasing studies had been reported that *S. suis* gradually showed resistance to these drugs, especially macrolides, sulfonamides and tetracyclines. The resistance rate was as high as 80~100%, and even multiplex resistant was detected [2,3,4]. Therefore, it is necessary to explore new sensitive drugs with reasonable dosage regimens for controlling the occurrence of drug resistance.

Aditoprim (ADP) is an antibacterial synergist developed in the 1980s. Early plasma pharmacokinetics study on swine indicated that ADP had the characteristics of wide apparent volume of distribution, long half-life and high bioavailability compared with other antibacterial synergists [5]. Additionally, it had a strong sensitivity to *S. suis* [6]. And early studies reported ADP had no obvious accumulation in animals [7,8] and had little genotoxicity and teratogenicity [9,10]. Therefore, it is feasible to develop a suitable preparation and formulate a reasonable dosage regimen for the treatment of infections caused by sensitive bacteria (particularly *S. suis*).

As a practical dosage form, injection has a series of advantages, such as accurate dose, rapid efficacy, high bioavailability and insensitivity towards digestive enzymes or food. According to European Pharmacopoeia and Chinese Veterinary Pharmacopoeia, the quality and stability of injections should be tested. The preliminary quality evaluations include appearance of injection, content, pH value, bacterial endotoxin and so on. Besides, the hemolysis and irritation research of injection are significant [11].

The dosage regimen is the basis of clinical medication. The pharmacokinetics-pharmacodynamics (PK-PD) integration model is a powerful tool widely used for formulating rational dosage regimen. The PK-PD model concerns on the relation between PK and PD, which can accurately understand the effect fluctuations with drug concentration and time, so as to formulate reasonable dosage regimen adapted to in vivo drug metabolism [12]. Ex vivo PK-PD model is the most widely used method to illustrate the relationship between drug concentration and ex vivo pharmacodynamic data. This model obtains PK data through determination of collected biologic samples and further data processing by pharmacokinetic software [13]. The ex vivo PK-PD model is of great importance in prodrug screening, drug candidate evaluation, novel dosage form development, personalized medicine and so on. However, it is difficult to figure out the drug concentration of infection tissues practically. And collecting tissue cage fluid or tissue homogenate is primary approach previously. For the infection of respiratory tract pathogens, the drug concentration measured by the above methods is different from the accurate value within lung epithelial tissue, where bacteria colonize. Therefore, ultrafiltration sampling technique, swab method and bronchoalveolar lavage method were put forward to improve accuracy [14,15,16]. Among them, swab method is hard to get access to respiratory tract pathogen samples. And the implementation of ultrafiltration is complicated. In contrast, bronchoalveolar lavage method can exactly collect bronchoalveolar lavage fluid (BALF) under narcotism assisted by convenient equipment [17,18,19]. It was reported that bronchoalveolar lavage method was more feasible for estimating precise drug concentrations, which was crucial to subsequent quantitative analysis [20].

In this paper, ADP injection was prepared and its pharmaceutical properties were evaluated. To carry out PK-PD modeling study of the injection against *S. suis* infection in pigs, bronchoalveolar lavage technique was applied to collect alveolar fluid samples. And further optimization of its clinical dosage regimen was deduced accordingly. More importantly, it is the first time to combine bronchoalveolar lavage technique and PK-PD modeling for investigating antibacterial agent. This study supported further development of ADP as a practical antibacterial injection, which provided an alternative for clinical treatment of *S. suis* infection in pigs.

## 2. Materials and Methods

### 2.1. Chemicals and Reagents

ADP standard (99% purity; 20 May 2013) and ADP API (98% purity; 20 December 2015) were obtained from Institute of Veterinary Medicine, Huazhong Agricultural University (Wuhan, China). Atropine sulfate injection (5 mL: 25 mg; 1 September 2015) was purchased from Sichuan Hengtong Animal Pharmaceutical Co., Ltd. (Neijiang, China). Propofol injection (10 mL: 100 mg; 1A150516) was bought from Guangdong Jiabo Pharmaceutical Co., Ltd. (Qingyuan, China). Sodium chloride injection (500 mL: 4.5 g; 1608090904) was purchased from Wuhan Binhu Shuanghe Pharmaceutical Co., Ltd. (Wuhan, China). Glycol propylene, hydrochloric acid and disodium hydrogen phosphate were obtained from Sinopharm Chemical Reagent Co., Ltd. (Shanghai, China). Fetal calf serum was bought from Zhejiang Tianhang Biological Technology Co., Ltd. (Huzhou, China). Tryptic soy broth (TSB) and tryptose soya agar (TSA) were purchased from Qingdao Hopebio Technology Co., Ltd. (Qingdao, China).

### 2.2. Bacteria

*S. suis* CVCC 607 was purchased from China Institute of Veterinary Drugs Control (Beijing, China). *E. coli* ATCC 25922 was donated by Pharmacology Laboratory of South China Agricultural University (Guangzhou, China).

### 2.3. Animals

Fifteen healthy castrated male crossbred (Duroc × Largewhite × Landrace) pigs with average weight of 20.0 ± 2.5 kg were provided by Livestock and Poultry Breeding Centre of Hubei Province (Wuhan, China). Before the experiment, the pigs were given a 7 days’ acclimatization within a stable environment (25 ± 2 °C and 45–65% relative humidity) with free intake of water and basal feed. And the animals were euthanized by intravenous injection of 100 mg/kg sodium pentobarbital (Euthanimal, Alfasan, Woerden, The Netherlands) after the end of the experiment [21,22]. All the animal experimental protocols complied with the guidelines of Laboratory Animal Use and Care Committee in Hubei Science and Technology Agency and were approved by the Animal Experiment Ethical Inspection of Laboratory Animal Center, Huazhong Agricultural University, Wuhan, China (HZAUSW-2018-021, 1 October 2018).

### 2.4. Preparation of ADP Injection

For preparation of 100 mL aditoprim injection, 10 g ADP was added into a mixed solvent (30 mL water and 30 mL propylene glycol) at room temperature to make a suspension solution. Then, 16 mL 10% hydrochloric acid solution was introduced to the solution under a water bath of 45 °C. After about 10 min mild agitation, ADP was fully dissolved. And 1% disodium hydrogen phosphate solution was dropwise added to adjust the pH value to 6.5 at room temperature. Finally, 0.18 g ethylparaben was dissolved into the solution. And the system was filled with water to the volume of 100 mL.

### 2.5. Quality Evaluation

The quality evaluations, such as pharmaceutical properties (appearance, specifications, pH value and drug content), safety (hemolysis and injection site irritation) and stability of ADP injection were investigated. The methods and results were exhibited in Appendix A, which proved to be a suitable preparation for subsequent study.

### 2.6. Pharmacodynamic Study

#### 2.6.1. Determination of MIC, MBC, MPC and PAE

The minimum inhibitory concentration (MIC) of ADP against *S. suis* CVCC 607 in broth medium was determined by microdilution method following CLSI (Clinical and Laboratory Standards Institute) protocol VET01 A4, 2013. And *E. coli* ATCC 25922 was used as a quality control strain. First, 100 μL bacterial suspension (10^6^ CFU/mL) was added to each well of 96-well plates. Then, 100 μL broth medium with serial dilution of ADP was introduced into above wells, respectively. And the final drug concentration ranged from 128 μg/mL to 0.25 μg/mL. After a 24 h incubation of the plates within 5% CO_2_ at 37 °C, the lowest ADP concentration of clear wells was determined as the MIC. To evaluate the minimal bactericidal concentration (MBC), 100 μL suspension taken from above MIC wells was subsequently diluted 10-fold with TSB broth medium. And 10 μL diluent was spread on MH agar plates to calculate colony-forming units after a 24 h incubation at 37 °C. The lowest concentration of ADP which reduced bacterial density by 99.9% was considered as MBC. For determine the ex vivo MIC and MBC, above broth medium was replaced by alveolar fluid (collected from the control pigs) [23].

The mutant prevention concentration (MPC) of ADP was detected by agar plate count method. 100 μL bacterial suspension of *S. suis* CVCC 607 (10^10^ CFU/mL) was inoculated on each agar plate containing serial concentration of ADP (1MIC, 2MIC, 4MIC, 8MIC, 16MIC, 32MIC and 64MIC) and then cultured at 37 °C for 72 h, respectively [24]. The lowest ADP concentration on the plate without bacterial growth was identified as MPC.

For detecting post-antibiotic effect (PAE), 100 μL ADP solution containing different amount of drug was added into 900 μL bacterial suspension of *S. suis* CVCC 607 (10^7^ CFU/mL) to give a final concentration of 1MIC, 2MIC and 4MIC, respectively. After exposure for 1 or 2 h, ADP was removed by centrifugation (5000 r/min, 3 min × 3). The treated bacteria were re-incubated in fresh broth medium for another 24 h, and quantification of the bacteria was measured by agar count plate method. The time variation for ADP-treated *S. suis* CVCC 607 to increase in number by 1 log_10_ minus the identical assessment for non-treated *S. suis* CVCC 607 was determined as the PAE [25]. All assessments were repeated in triplicate.

#### 2.6.2. Establishment of In Vitro and Ex Vivo Growth Inhibition Curves

The in vitro growth inhibition curves were evaluated in TSB broth medium. The 10^6^ CFU/mL bacteria solution of *S. suis* CVCC 607 was incubated together with different concentrations of ADP in TSB broth medium (0, 1/2, 1, 2, 4, 8, 16 and 32 MIC) at 37 °C for 24 h. The colony-counting method was adopted to calculate bacterial numbers of the culture at 0, 0.5, 1, 1.5, 2, 3, 4, 6, 8, 12, 24, 36 and 48 h. And the ex vivo growth inhibition curves were assessed in alveolar fluid as culture medium. The BALF of pigs was collected periodically after intramuscular injection with ADP injection and concentrated 10 times by lyophilization. *S. suis* CVCC 607 was incubated in condensed alveolar fluid with a concentration of 10^6^ CFU/mL at 37 °C for 24 h. Each test was carried out in triplicate. At last, both growth inhibition curves of ADP against *S. suis* CVCC 607 were drawn with time as abscissa and logarithm of bacteria amount as ordinate, respectively.

### 2.7. Pharmacokinetic Study

Twelve pigs were randomly distributed into two groups (*n* = 6). Group 1 were healthy pigs, and group 2 were *S. suis*-infected pigs. For group 2, each pig was inoculated with 1 mL bacterial suspension of *S. suis* CVCC 607 (1.2 × 10^9^ CFU/mL) subcutaneously. And subsequent experiment was carried out after some typical streptococcosis symptoms (coughed, corneal flushed, breathing frequency increased, inappetence, high temperature, spiritual malaise, joints swelling, central nervous system signs) were observed [23]. Blank samples of blood and bronchoalveolar lavage fluid were collected before ADP administration. Then, ADP injection was administered to the hip of pigs by intramuscular injection at a dose of 5 mg/kg according to the previous research. Blood samples were taken at 0, 0.083, 0.167, 0.25, 0.5, 0.75, 1, 1.5, 2, 3, 4, 6, 8, 10, 12 and 24 h. And bronchoalveolar lavage fluid samples were gathered at 0, 0.5, 1, 1.5, 2, 3, 4, 6, 8, 12, 24, 36 and 48 h.

For collecting bronchoalveolar lavage fluid, the premedication with atropine sulfate injection at a dose of 0.05 mg/kg b.w. intramuscularly and propofol injection at a dose of 6 mg/kg b.w. intravenously was applied to achieve anesthesia during BALF sampling. Assisted by real-time synchronous monitor, electronic fiberoptic bronchoscope was inserted into the trachea and 50 mL 25 °C saline was infused into the fourth-grade bronchus of anesthetized pig. 60 s later, the above bronchoalveolar lavage solution was extracted by an air pump and collected. After a further centrifugation (800 r/min) at 4 °C for 10 min, the precipitates were discarded to obtain BALF. And the content of urea nitrogen in BALF and plasma sample at the same time point was detected to calculate the dilution multiple of drug concentration in alveolar fluid [15].

Plasma and bronchoalveolar lavage fluid samples were assayed by Waters 2695 series HPLC (Waters Corporation, Milford, CT, USA) with a UV detector. 1 mL plasma or bronchoalveolar lavage fluid sample was mixed with 4 mL extraction solvent (ethyl acetate:methanol = 95:5), and the mixture was vortexed for 3 min. After centrifugation at 10,000 r/min and 4 °C for 10 min, the supernatant was extracted. The residual solution was extracted repeatedly. The entire supernatant was evaporated to dry at 45 °C under nitrogen. The obtained residue was reconstituted in 1 mL methanol. After filtered through a 0.22 μm membrane, 40 μL reconstituted sample was applied to conduct HPLC detection. A ZORBAX SB-C_18_ column (250 mm × 4.6 mm i.d., 5 μm; Agilent Technologies, Santa Clara, CA, USA) was utilized to separate at 25 °C. The mobile phase composed of 0.06% trifluoroacetic acid (TFA) and acetonitrile from Sigma-Aldrich Chemicals (Poole, Dorset, UK) for gradient elution. And the detection wavelength was 240 nm.

For validation of the HPLC method, the limit of detection (LOD) and limit of quantification (LOQ) of ADP either in plasma or in bronchoalveolar lavage fluid was 0.04 μg/mL and 0.08 μg/mL, respectively. The calibration curves of ADP in plasma and bronchoalveolar lavage fluid were both ranged from 0.08 μg/mL to 1 μg/mL with regression coefficients (*r*^2^) greater than 0.99. The mean recovery of ADP either in plasma or in bronchoalveolar lavage fluid was higher than 80% and the variation coefficient was smaller than 10%. These validation results were complied with the residue guidelines of the Veterinary Pharmacopoeia of the Department of Agriculture and the Pharmacopoeia of the United States [26].

### 2.8. PK-PD Modeling and Dosage Regimen

ADP concentration-time data in plasma and alveolar fluid for individual pig were analyzed by two compartment model analysis (WinNonlin, version 5.2.1, Pharsight Corporation, Mountain View, CA, USA) to obtain the pharmacokinetic parameters. The relation of drug concentration and time was also described through the semi-logarithmic drug concentration-time curve. For PK-PD integration, the parameter AUC_0–24 h_/MIC revealed ADP in porcine alveolar fluid. For PK-PD modelling, AUC_0–24 h_/MIC and antibacterial effect (*E*) from ex vivo bacterial growth inhibition curves were introduced to the *Sigmoid E_max_* equation:

$$E=E_{\max}-\frac{(E_{max}-E_{0})\cdot C^{N}}{C^{N}+EC_{50}{}^{N}}$$

Note: *E* is the antibacterial effect measured as the change on the logarithmic reduction values of bacterial count (log_10_ CFU/mL) in alveolar fluid sample after 24 h compared to the initial value; *E*_0_ is the antibacterial effect measured as the maximum on the logarithmic reduction values of bacterial count in alveolar fluid sample after 24 h compared to the initial value; *E_max_* is the antibacterial effect measured as the change on the logarithmic reduction values of bacterial count in blank alveolar fluid after 24 h compared to the initial value; *EC*_50_ is the AUC_0–24 h_/MIC value achieving 50% of the maximal antibacterial effect; *C* is the AUC_0–24 h_/MIC of ex vivo PK-PD modeling; *N* is the Hill coefficient which describes the steepness of the AUC_0–24 h_/MIC-effect curve.

The antibacterial effect of ADP against *S. suis* CVCC 607 was quantified at three levels by calculation of AUC_0–24 h_/MIC for bacteriostatic action, bactericidal action and bacterial eradication. When E = 0 (no change in bacterial count after 24 h incubation), E = −3 (99.9% reduction of the original inoculum count after 24 h incubation) and E = −4 (99.99% reduction of the original inoculum count after 24 h incubation), the corresponding AUC_0__–24 h_/MIC values were defined as bacteriostatic action, bactericidal action and bacterial eradication, respectively.

After determining the AUC_0–24 h_/MIC values of different antibacterial effect, the doses were calculated by following formula:

$$\mathrm{Dose}=\frac{({\mathrm{AUC}}_{24}/\mathrm{MIC})\times \mathrm{MIC}\times \mathrm{CL}}{fu\times F}$$

Note: CL is the clearance rate; *fu* is the free fraction of drug; F is the bioavailability; MIC is the MIC of target strain. In this study, the CL/F was obtained in the pharmacokinetic study; MIC was 2 μg/mL; the protein binding rate of ADP in alveolar fluid was below 1%, thus the *fu* was considered as 1 (illustrated in Appendix B).

The MlxPlore software (version1.1.0, Lixoft, Orsay, France) was applied to predict the bacterial growth at different dosage regimen, which could deduce the optimal dosing interval and dosage regimen.

## 3. Results

### 3.1. Pharmacodynamic Study

#### 3.1.1. The MIC, MBC, MPC and PAE of ADP against *S. suis* CVCC 607

The MIC of ADP against *S. suis* CVCC 607 in TSB broth medium and porcine alveolar fluid were both 2 μg/mL. And the MBC in TSB broth medium and porcine alveolar fluid were both 4 μg/mL. The MPC of ADP was 12.8 μg/mL. The PAE of ADP against *S. suis* CVCC 607 was presented on Table 1. The results indicated that the PAE of ADP positively associated with the concentration of ADP. Along with the rise of drug concentration, the PAE increased gradually. Moreover, the PAE also presented positive correlation with the exposure time of *S. suis* CVCC 607 in drug solution.

#### 3.1.2. The In Vitro and Ex Vivo Antimicrobial Activity

**Figure 1:**
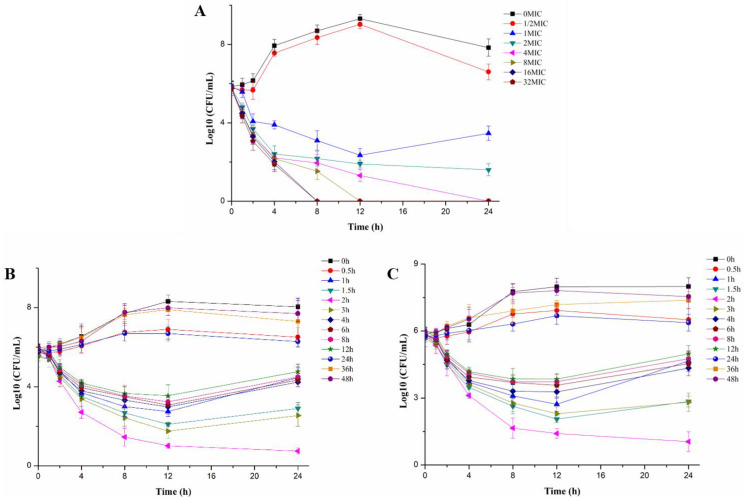
The in vitro growth inhibition curves of ADP in TSB broth medium (**A**) and ex vivo growth inhibition curves in alveolar fluid from healthy pigs (**B**) and infected pigs (**C**) against *S. suis* CVCC 607.

In vitro and ex vivo growth inhibition curves of ADP against *S. suis* CVCC 607 were illustrated in Figure 1. The antibacterial activity enhanced gradually with the increase of ADP concentration, which exhibited the characteristic of concentration-dependent drug. The high ADP concentration could reduce the number of bacteria or even kill bacteria rapidly. However, the bacteria incubated within low drug concentration would recover growth after 12 h. The ex vivo growth inhibition curves in alveolar fluid of healthy pigs and infected pigs against *S. suis* CVCC 607 were analogous. Consequently, the preferential PK-PD parameter was AUC/MIC.

The *E_max_*, *E*_0_ and bacterial logarithmic reduction values were deduced on the basis of ex vivo growth inhibition curves. The pharmacodynamic model of WinNonlin software was applied to simulate the relation between bacterial logarithmic reduction values and AUC_0–24 h_, and obtain the *EC*_50_ and *N* values. The AUC_0–24 h_ was evaluated by 24 × *C_max_* (*C_max_* was the average value of ADP in alveolar fluid at different time points; 24 referred to the incubation time of bacteria). The *EC*_50_ and *N* values of healthy group and infected group were 25.00, 2.24 and 24.00, 2.48, respectively (Table 2).

### 3.2. Pharmacokinetic Study

**Figure 2:**
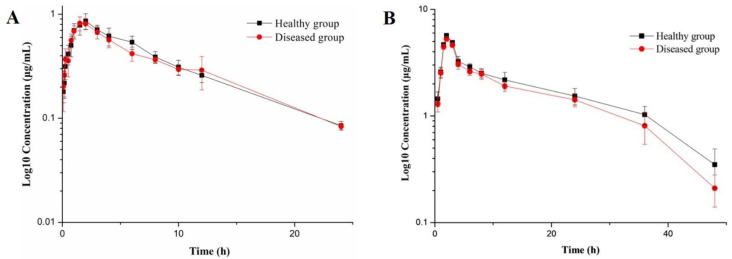
The semi-logarithmic plot for the concentration of ADP in plasma (**A**) and alveolar fluid (**B**) after intramuscular administration of ADP injection at 5 mg/kg b.w. (*n* = 6).

The semi-logarithmic drug concentration-time curves were plotted according to the concentration of ADP in plasma and alveolar fluid (as shown in Figure 2). And the PK parameters were represented in Table 3. The SPSS software was applied to analyze the PK parameters of healthy group and infected group in plasma and alveolar fluid, respectively. It was found that all parameters had no significant difference between healthy group and infected group, which indicated the pharmacokinetic process in plasma and alveolar fluid of ADP in pigs infected by streptococcus suis was similar to that of the healthy group. On the other hand, significant differences were observed between the ADP concentrations in plasma and in alveolar fluid, and the latter was obviously higher than that in plasma. The values of AUC and C_max_ in alveolar fluid were higher than those in plasma, while the situation for CL/F was opposite.

### 3.3. PK-PD Modeling and Dosage Regimen

Based on the above pharmacokinetic data, the Sigmoid E_max_ model parameters of ADP in alveolar fluid were showed in Table 2. When E was set as 0, −3 and −4, the AUC_0__–24 h_/MIC in the healthy group and the infected group were 17.07, 37.38, 53.98 and 17.04, 35.54, 51.27, respectively.

The doses of ADP injection under three levels of antibacterial effect were illustrated in Table 4. Due to clinical medication mainly for diseased animals, the results of the infected group were chosen as recommend doses ultimately.

**Figure 3:**
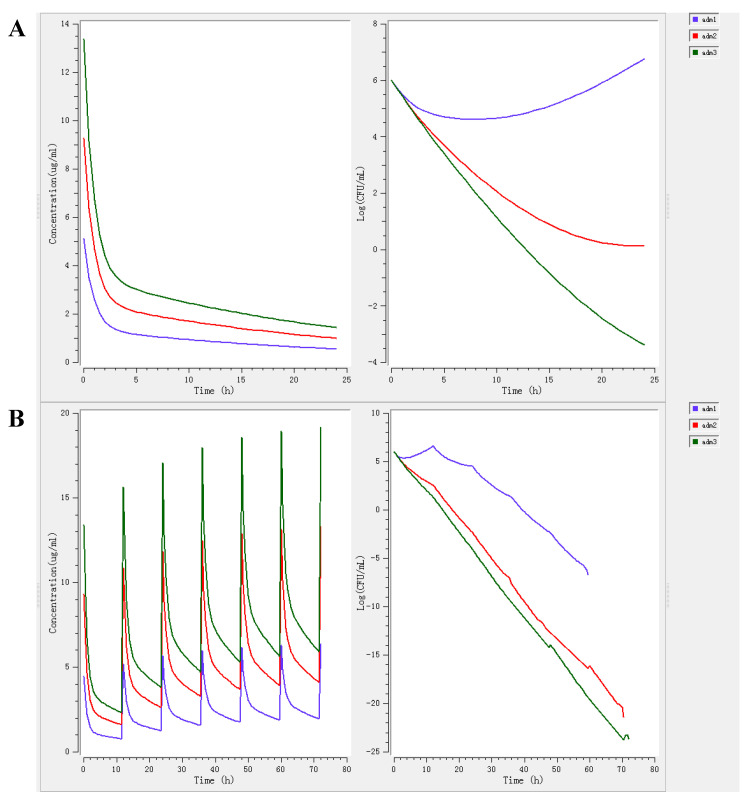
The MlxPlore simulated diagrams of ADP injection with different dosage regimen (adm1: 1.96 mg/kg b.w.; adm2: 4.10 mg/kg b.w.; adm3: 5.91 mg/kg b.w.): (**A**): the drug concentration and bacterial growth in 24 h after a single dose injection; (**B**): the concentration and bacterial growth in 72 h after a multidose injection with an interval of 12 h.

The MlxPlore simulated diagrams of ADP injection at different dosage regimen were shown in Figure 3. Each simulated diagram consisted of two parts: drug concentration change (left) and bacterial growth (right). For a daily single dose treatment, the bacterial growth at a dose of 1.96 mg/kg was inhibited during 0~10 h, but the bacteria continued to grow during 10~24 h (Figure 3A). For a multi-dose administration in 3 days with an interval of 12 h, the bacterial growth at a dose of 1.96 mg/kg was inhibited sustainedly. And the higher dose of 4.10 mg/kg or 5.91 mg/kg had a better antibacterial effect (Figure 3B). Additionally, the PAE of ADP exhibited a shorter time. In order to avoid the emergence of bacteria resistance effectively, the optimal choice was intramuscular injection twice a day. Accordingly, for the dose of 1.96 mg/kg ADP, the dosage regimen of 3~5 days with an interval of 12 h was used to inhibit bacteria growth and prevent the infection of *S. suis*. The dosage regimen of 3~5 days with an of interval 12 h at 4.10 mg/kg or 5.91 mg/kg was adopted to treat the infection of *S. suis*.

## 4. Discussion

ADP and trimethoprim (TMP) belong to alkaline sulfonamide synergists, which are difficult to dissolve in water. Therefore, to create an acidic condition (pH 3.5~5.5), lactic acid was added as a pH modifier to dissolve drug in TMP injection. Similarly, to increase the drug content and avoid irritation reactions of ADP injection, hydrochloric acid solution was screened as the pH modifier in this study. And disodium hydrogen phosphate was used to maintain the pH of injection. The drug content of ADP injection reached 10% and its pH value was about 6.20. Meanwhile, the mixed solvent composed of propylene glycol and water was applied as the cosolvent of ADP injection in order to improve the quality and stability of ADP injection. Eventually, the obtained injection exhibited no hemolysis and irritation to target animals, which preliminarily illustrated its reliable safety. The results of accelerated test and long-term test demonstrated the good performance on stability of ADP injection.

The MIC of ADP against numerous *S. suis* strains (76) had been studied previously, and its MIC_50_ and MIC_90_ value was determined as 2 μg/mL and 32 μg/mL, respectively [6]. It indicated that ADP had a good antibacterial activity against *S. suis*, and could be considered to treat the infection of *S. suis.* The MIC and MBC of *S. suis* CVCC 607 was 2μg/mL and 4μg/mL respectively. The above values were just within the range of MIC_50_, thus the *S. suis* CVCC 607 was adopted for the PK-PD study. Furthermore, *S. suis* CVCC 607 belonged to *S. suis* serotype 2, which was considered to have the strongest pathogenicity with wide epidemicity and serious harm to animals [27,28]. Thus, *S. suis* CVCC 607 was adopted to establish the *S. suis* infected model and conducted the pharmacodynamics research in this study.

Due to the complicated constituents of test systems, the matrix effect is important to investigate MIC of antibacterial agents. There are not only different formulas of classic culture mediums, but also various proteins and cytokines of biological samples, which directly influence the inhibition effect to bacteria [29]. To get rid of matrix effect, porcine alveolar fluid was utilized to evaluate a more precise MIC result of ADP against *S. suis* CVCC 607 in this paper. And the MIC in TSB broth medium and alveolar fluid were both 2 μg/mL. In fact, other factors (such as surfactant content and macrophage quantity) may also affect the accuracy of MIC value [30]. Therefore, it is necessary to make further exploration of matrix effect for a more specifical and accurate antibacterial efficiency in alveolar fluid. For example, Rodvold et al. exploited microdialysis technique to assess the concentration of antimicrobial drugs in lung, which provided an alternative to bronchoalveolar lavage method [31].

In vitro and ex vivo growth inhibition curves of ADP against *S. suis* CVCC 607 revealed that the antibacterial ability of ADP attributed to concentration-dependent antibacterial drug. And the main PK-PD parameter of these antibacterial drugs was AUC_24 h_/MIC or C_max_/MIC [32,33,34]. Therefore, the PK-PD integration parameter of AUC_24 h_/MIC was selected in this study.

Although most pharmacokinetics studies collected plasma samples to establish the relationship between drug concentration and antibacterial effect, it is more significant to study the distribution and dynamic change of drug concentration at infection sites. For the diseases caused by respiratory tract pathogens such as *S. suis*, lots of bacterial colonization sites are lung epithelial tissues. However, it was difficult to determine the drug concentration in lung epithelial tissue fluid. The available methods to detect the drug concentration of lung were collecting tissue cage fluid [13] and lung homogenate [35]. Nevertheless, the drug concentration of lung measured by above methods was different from the drug concentration of lung epithelial tissue fluid [36]. In order to accurately evaluate the drug concentration at infection sites of lung, researchers put forward several reasonable and advanced research methods. One was swab wiping method [14,37], and the other was bronchoalveolar lavage method [15,38]. And it had been proved that bronchoalveolar lavage method was a suitable method for bacteriological and cytological study [20]. In addition, the ultrafiltration sampling techniques was also adopted to collect the drug of bronchial fluid [16,39,40]. And a relatively high requirement of apparatus hindered its wide application. In this study, the concentration of ADP in alveolar fluid was determined by collecting BALF. It was reported that 50 mL physiological saline for bronchoalveolar lavage could not only ensure effective sampling time, but also prevent animal from shock or death [15]. The dilution multiple of drug concentration in alveolar fluid after lavage was assessed by comparing the content of urea nitrogen in bronchoalveolar lavage fluid and plasma sample at the same time point. The results showed that the concentration of urea nitrogen in plasma was about 10 times of that in bronchoalveolar lavage fluid, which indicated the accurate drug concentration in alveolar fluid was 10 times of that in bronchoalveolar lavage fluid.

After administrated by intramuscular injection, plasma and bronchoalveolar lavage fluid were collected to determine the concentration of ADP. In plasma, the concentration of ADP could be detected in 0~24 h, while in alveolar fluid it could be detected during 0 ~48 h. The peak concentration (C_max_) of ADP in plasma was not only under one sixth of that in alveolar fluid, but also lower than its MIC against *S. suis* CVCC 607. Therefore, the pharmacokinetics of ADP in alveolar fluid was studied in this paper. Moreover, early pharmacokinetic studies of ADP only focused on the drug concentration in plasma, and no pharmacokinetic data of ADP in alveolar fluid were reported.

In the early study on the pharmacokinetics of ADP, Riond used the 3-month-old and 6-month-old healthy pigs as experimental animals to obtain plasmatic pharmacokinetic parameters after intravenous injection and oral administration of 5 mg/kg ADP, respectively [5]. The results showed the AUC of ADP in 3-month-old and 6-month-old healthy pigs was 5.92 ± 0.81 h·μg/mL and 11.51 ± 1.07 h·μg/mL for intravenous injection respectively; the corresponding C_max_ was 2.13 ± 0.32 μg/mL and 3.44 ± 0.94 μg/mL respectively. For oral administration, the AUC of ADP in 3-month-old and 6-month-old healthy pigs was 4.10 ± 0.73 h·μg/mL and 6.12 ± 1.09 h·μg/mL respectively; the corresponding C_max_ was 0.21 ± 0.03 μg/mL and 0.30 ± 0.11 μg/mL respectively. Compared with the pharmacokinetic data obtained in this study, the AUC and C_max_ in plasma after intravenous injection and oral administration were significantly lower than that in alveolar fluid after intramuscular injection. It indicated the distribution of ADP in alveolar fluid was superior to that in plasma, which might have a greater effect for the treatment of *S. suis* infection. And the C_max_ of plasmatic drug in this study was between that of oral administration and intravenous injection, which might be affected by drug administration route. In addition, by combining ADP and sulfamethoxazole (SMZ) with a mass ratio of 1:5 into an injection, Wang et al. studied the antibacterial activity of this injection against *E. coli* in swine [25]. After intramuscular administration of ADP/SMZ at a single dose of 5/25 mg/kg b.w., the C_max_ and T_max_ of ADP in the plasma of healthy pigs was 0.89 ± 0.18 μg/mL and 2.17 ± 0.37 h, respectively. These results were similar with the plasma concentration of ADP in this paper, which indicated a relatively quick absorption process of ADP happened in pigs. Moreover, to treat swine colibacillosis due to *E. coli*, a dosage of 3.45/17.25 mg/kg ADP/SMZ by intramuscular injection daily for 3 consecutive days was concluded by PK-PD modeling. The administration dosage and frequency of ADP/SMZ injection were superior to our ADP injection, which might be attributed to the combined antibacterial activity of SMZ and ADP and different diseases (*S. suis* caused pulmonary infection and *E. coli* caused intestinal infection).

The basic integration models of PK-PD include linear model, log-linear model, maximum effect model (E_max_ model), Sigmoid E_max_ model and β-function model. Among them, the Sigmoid E_max_ model was the most widely used tool. In this study, the Sigmoid E_max_ model was adopted to integrate the pharmacokinetics of ADP and ex vivo pharmacodynamics. Combined with dose formula, the dose for bacteriostatic action, bactericidal action and bacterial eradication of the infected group were 1.96, 4.10, 5.91 mg/kg, respectively. Additionally, mutant selection window (MSW) theory predicts the occurrence of resistant bacteria, especially when the drug concentration is slightly higher than the MIC [41]. Combined with above pharmacokinetic data, ADP concentration in two-thirds of 24 h was slightly higher than the MIC after a single dose, which would cause a risk of drug resistance. Hence, by multi-dose administration of ADP with 12 h interval, it not only achieved rapid treatment effect against *S. suis* infection, but also reduced the generation of drug-resistant bacteria. Furthermore, this dosage regimen coincided with the deduced results of MlxPlore. Ultimately, the dosage regimen of ADP injection for intramuscular administration with an interval of 12 h was considered as a more rational treatment protocol.

## 5. Conclusions

A novel ADP injection was prepared successfully for treating *S. suis* infection of pigs, and quality assessment preliminarily indicated its reliable quality and stability. What’s more, the bronchoalveolar lavage fluid method was first applied to evaluate accurate ADP concentration at infection site in this study, which not only provided valid data to formulate a more scientific and rational dosage regimen, but also helped to reduce the occurrence of drug-resistant bacteria. Furthermore, PK-PD modeling predicted a dosage regimen of 4.10 mg/kg every 12 h for 3 days should be effective for the treatment of *S. suis* infection in pigs. Consequently, this ADP injection with a multi-dose protocol would be a promising antimicrobial product for efficient treatment of *S. suis* infection of pigs.

## Figures and Tables

**Table 1:** The PAE of ADP against *S. suis* CVCC 607.

Concentration (μg/mL)	PAE (h)	
	Exposed for 1 h	Exposed for 2 h
1 MIC	0.25	0.53
2 MIC	0.71	0.93
4 MIC	1.09	1.28

**Table 2:** The Sigmoid *E_max_* model parameters of ADP in alveolar fluid after intramuscular administration of ADP injection at 5 mg/kg b.w.

Parameters	Units	Healthy Group	Infected Group
*E_max_*	Log_10_ CFU	2.17	2.11
*E*_0_	Log_10_ CFU	−5.10	−4.93
*E_max_*-*E*_0_	Log_10_ CFU	7.27	7.04
*EC*_50_	h	25.00	24.00
*N*	-	2.24	2.48
Bacteriostatic (E = 0)	h	17.07	17.04
Bactericidal (E = −3)	h	37.38	35.54
Eradication (E = −4)	h	53.98	51.27

Note: *E_max_* is the antibacterial effect measured as the change on the logarithmic reduction values of bacterial count in blank alveolar fluid after 24 h compared to the initial value; *E*_0_ is the antibacterial effect measured as the maximum on the logarithmic reduction values of bacterial count in alveolar fluid sample after 24 h compared to the initial value; *EC*_50_ is the AUC_0–24 h_/MIC value achieving 50% of the maximal antibacterial effect; *N* is the Hill coefficient which describes the steepness of the AUC_0–24 h_/MIC-effect curve; *E* is the antibacterial effect measured as the change on the logarithmic reduction values of bacterial count (log_10_ CFU/mL) in alveolar fluid sample after 24 h compared to the initial value.

**Table 3:** The PK parameters of ADP in plasma and alveolar fluid after intramuscular administration of ADP injection at 5 mg/kg b.w. (*n* = 6).

Parameters	Healthy Group		Infected Group	
	Plasma	Alveolar Fluid	Plasma	Alveolar Fluid
AUC (h·μg/mL)	8.82 ± 0.94	95.38 ± 11.74	9.33 ± 1.52	86.75 ± 8.26
CL/F (mL/h)	573.07 ± 71.81	52.42 ± 3.03	545.99 ± 72.59	57.64 ± 2.56
T_max_ (h)	1.96 ± 0.34	1.96 ± 0.14	1.75 ± 0.29	1.95 ± 0.15
C_max_ (μg/mL)	0.80 ± 0.11	5.45 ± 0.47	0.77 ± 0.07	5.10 ± 0.17
A (μg/mL)	−30.49 ± 54.07	1.08 ± 0.01	−49.80 ± 150.66	1.10 ± 0.03
Β (μg/mL)	0.72 ± 0.14	0.03 ± 0.01	0.54 ± 0.18	0.04 ± 0.02
T_1/2α_ (h)	1.25 ± 0.63	0.64 ± 0.15	1.46 ± 0.97	0.62 ± 0.09
T_1/2β_ (h)	7.95 ± 0.81	5.32 ± 0.74	11.09 ± 2.78	5.33 ± 0.49

Note: AUC, area under drug concentration-time curve; CL/F, body clearance corrected for bioavailability; T_max_, time to achieve the maximum alveolar concentration; C_max_, the maximum alveolar concentration; A, intercept of absorption phase equation; B, intercept of elimination phase equation; T_1/2α_, absorption half-life; T_1/2__β_, elimination half-life.

**Table 4:** The doses of ADP injection for different antibacterial effects.

Effect (E)	Healthy Group	Infected Group
Bacteriostatic action (E = 0)	1.79 mg/kg b.w.	1.96 mg/kg b.w.
Bactericidal action (E = −3)	3.92 mg/kg b.w.	4.10 mg/kg b.w.
Bacterial eradication (E = −4)	5.66 mg/kg b.w.	5.91 mg/kg b.w.

**Figure A1:**
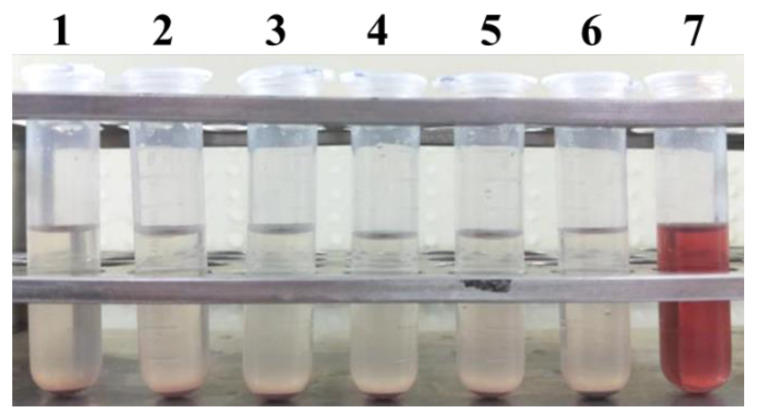
The hemolysis test of ADP injection within 37 °C at 3 h.

**Figure A2:**
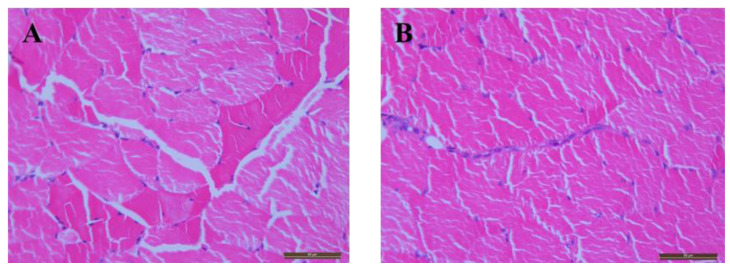
The typical pathological section of injection sites: (**A**): ADP injection; (**B**): saline (×400).

## Data Availability

Data can be requested by contacting the corresponding author.

## Appendix A. Quality Evaluation of ADP Injection

### Appendix A.1. Appearance, Specifications, pH Value and Content Determination

The color and clarity of ADP injection were observed. The specification referred to the amount of ADP in injection. The pH value was detected by pH meter according to the instruction. The drug content of ADP injection was determined by high performance liquid chromatography (HPLC).

The basic pharmaceutical properties of ADP injection were investigated following Chinese Veterinary Pharmacopoeia (2020). This injection was a clear yellow liquid with 10 g ADP distributing in every 100 mL solution uniformly. The drug content of the injection was 99.35~100.40%, which conformed to corresponding pharmacopoeia requirement (95.00~105.00%). The pH value of the injection was about 6.20. The identification test results illustrated that the maximum absorption peak of ADP (283 nm) appeared in both UV spectrums of the injection and ADP standard solution. Meanwhile, the retention time of ADP generated by ADP injection was the same as that of standard solution in HPLC chromatograms.

### Appendix A.2. Hemolysis and Injection Site Irritation

On the basis of Chinese Veterinary Pharmacopoeia, the hemolysis of ADP injection was investigated by visual observation. First, 7 test tubes containing 2.5 mL 2% erythrocyte suspension were prepared separately. According to Table A1, different volume of physiological saline was added. Then, corresponding amount of injection was added into tube 1~5, while physiological saline and distilled water was added into tube 6 and tube 7 as the negative control and positive control, respectively. All the tubes were kept at 37 °C for 3 h to test hemolysis.

**Table A1:** The preparation of different solutions containing erythrocytes for hemolysis test.

Tube Number	1	2	3	4	5	6	7
2% erythrocyte suspension (mL)	2.5	2.5	2.5	2.5	2.5	2.5	2.5
Physiological saline (mL)	2.4	2.3	2.2	2.1	2.0	2.5	0
Distilled water (mL)	0	0	0	0	0	0	2.5
ADP Injection (mL)	0.1	0.2	0.3	0.4	0.5	0	0

Hemolysis and injection site irritation are important components of the safety and quality control indexes of injections. After 3 h incubation, the solution within tube 7 exhibited red transparently, while the solution in tube 1~6 was colorless clear liquid (Figure A1). And there was small amount of precipitation at the bottom of tube 1~6, which could be redispersed by gentle shaking. The results indicated that ADP injection had no hemolysis.

The injection site irritation of ADP injection in pig was observed by self-comparison method. The injection was administrated into the left gluteus medius of pigs at a dose of 0.05 mL/kg, and equal amount of physiological saline was injected into the right gluteus medius. Then clinical symptoms were monitored every six hours. After 24 h, pigs were necropsied to inspect internal abnormalities at injection sites. The muscle at injection site was also collected for histopathological examination using hematoxylin-eosin staining.

After intramuscular administration of ADP injection, the pig did not show any clinical symptom of pain or discomfort. And no pathological change of degeneration, necrosis, red and swollen, exudation or hyperemia was observed at the injection site. The pathological section of injection sites also showed no pathological change (Figure A2), which preliminarily manifested that ADP injection had no irritation to injection sites.

### Appendix A.3. Stability Studies

According to technical standards of veterinary drug stability testing, accelerated experiment and long-term experiment were carried out to evaluate the stability of ADP injection. During accelerated experiment, ADP injections were stored at 40 ± 2 °C and 75 ± 5% relative humidity (RH) for 6 months to inspect its property, content, pH value and visible particles periodically. The long-term experiment demanded the injections were stored at 25 ± 2 °C and 60 ± 5% RH for 12 months to study above indexes periodically.

As illustrated in Table A2, ADP injection showed no obvious change of color or clarity during stability investigation. The drug content varied from 99.77 ± 0.56% to 99.16 ± 0.71% in accelerated experiment, while the content changed between 99.77 ± 0.56% and 99.56 ± 0.60% in long-term experiment. The pH value of ADP injection held steady around 6.22. The fluctuation of its content and pH value was smaller than 5.00% and 1.00 respectively, which proved to be stable during product storage and transport preliminarily.

**Table A2:** The results of accelerated experiment and long-term experiment of ADP injection (*n* = 3).

Projects	Time	Color	Content (%)	pH	Visible Particles
Accelerated test	0 month	yellow	99.77 ± 0.56	6.21 ± 0.04	None
	1 month	yellow	99.64 ± 0.51	6.23 ± 0.04	None
	2 months	yellow	99.48 ± 0.50	6.22 ± 0.04	None
	3 months	yellow	99.45 ± 0.62	6.25 ± 0.03	None
	6 months	yellow	99.16 ± 0.71	6.23 ± 0.03	None
Long-term test	0 month	yellow	99.77 ± 0.56	6.21 ± 0.04	None
	3 months	yellow	99.67 ± 0.61	6.21 ± 0.04	None
	6 months	yellow	99.62 ± 0.59	6.21 ± 0.05	None
	9 months	yellow	99.63 ± 0.60	6.21 ± 0.04	None
	12 months	yellow	99.56 ± 0.60	6.22 ± 0.01	None

## Appendix B. Determination the Free Fraction of ADP in Alveolar Fluid

The free fraction of ADP in alveolar fluid was measured by equilibrium dialysis method according to previous protocols [42]. Dialysis tubes (MWCO = 2000 Da) containing 2 mL alveolar fluid were put into beakers with 47.8 mL saline, respectively. 0.2 mL saline solution with different amount of ADP was added to each beaker to obtain final drug concentration of 1, 10, 50 mg/L, respectively. Additionally, to estimate the equilibrium time, alveolar fluid was replaced by saline in above procedure. The sealed beakers were stirred mildly at 37 °C. After 72 h, alveolar fluid within dialysis tubes and dialysis solution in beakers were collected to assess the concentration of ADP by HPLC mentioned in Section 2.7. And the assessments were repeated in triplicate. The results indicated that the concentration of ADP in dialysis solution was above 99% of that in alveolar fluid, which illustrated the high proportion of free drug in alveolar fluid.
